# Supplementary material for: Splice-Junction-Based Mapping of Alternative Isoforms in the Human Proteome
Source: Cell Rep. Author manuscript; Available in PMC 2020 Jan 15. (PMC6961840; doi:10.1016/j.celrep.2019.11.026)

A

sp|O75592|MYCB2_HUMAN|ENSG00000005810|SE1|11340|chr13|77126542|77129230|−0|r17|T1
SLLVPVDNIFNASQGVR q value: 4.3e−05 Tr_novel:TRUE RefSeq_Novel:FALSE
Search result spec prec mz: 915.0034 Actual spec prec mz: 915.00342
Fragments matched per AA: 2.41 Proportion of top 20 peaks matched: 0.35

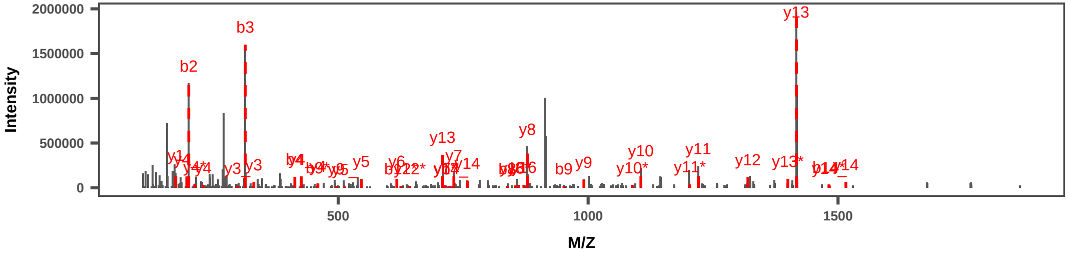

B

Scatterplot of predicted elution time
Fitting R2: 0.717
Novel peptide residual Z score: 0.778
Number of peptides: 3314

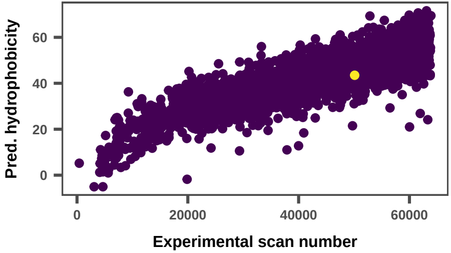

C

Distributions of residuals from best−fit line
of predicted RT vs Expt. scan number
Line: Z score of novel peptide
Z: 0.778

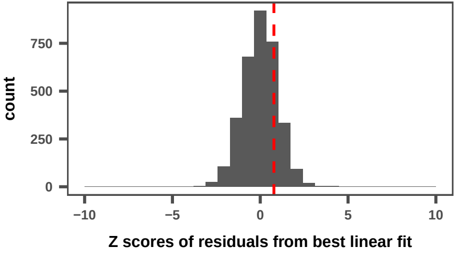

Supplement: 2 [file NIHMS1546469-supplement-2.zip › DF1/PXD009737/TestisTrypsin/TestisTrypsin_27_MYCBP2_SLLVPVDNIFNASQGVR.pdf]
